# Immunosuppression with cyclophosphamide favors reinfection with recombinant *Toxoplasma gondii* strains

**DOI:** 10.1051/parasite/2012193249

**Published:** 2012-08-15

**Authors:** L.A. Silva, G.P. Brandão, B.V. Pinheiro, R.W.A. Vitor

**Affiliations:** 1 Departamento de Parasitologia, Instituto de Ciências Biológicas, Universidade Federal de Minas Gerais (UFMG) Av. Antonio Carlos 6627 31270-901 Belo Horizonte, MG Brazil

**Keywords:** *Toxoplasma gondii*, recombinant strain, reinfection, cyclophosphamide, *Toxoplasma gondii*, souche recombinante, réinfection, cyclophosphamide

## Abstract

The aim of this study was to verify the effect of immunosuppression by cyclophosphamide (Cy) on susceptibility of BALB/c mice subjected to challenge with recombinant strains of *Toxoplasma gondii*. Animals were prime infected with the D8 (recombinant I/III) or the ME49 (type II) non-virulent strains, weekly immunosuppressed with Cy and challenged with the CH3 or EGS virulent strains (I/III). Parasites recovered from surviving mice were submitted to PCR-RFLP analysis to confirm co-infection. Prime-infection with the D8 strain conferred more protection against challenge with the CH3 and EGS strains when compared with ME49 prime infection. Cy treatment caused significant leukopenia in the infected mice, what probably favors reinfection after challenge. Reinfection was associated with increased levels of IgA. Otherwise, Cy-treated mice presented significantly lower IgA levels after challenge, suggesting involvement of this immunoglobulin on protection against reinfection. In conclusion, BALB/c mice susceptibility to reinfection by *T. gondii* is related to genetic differences among the strains used for primary and challenge infections. Alteration of the host’s immune integrity by Cy probably compromises the protection previously established by primary infection.

## Abstract (fr)

L’objectif de cette étude était de vérifier l’effet de l’immunosuppression par la cyclophosphamide (Cy) sur la susceptibilité de souris BALB/c soumises à la réinfection par des souches recombinantes de *Toxoplasma gondii*. Les souris ont été infectées par des souches non virulentes de *T. gondii* : D8 (recombinant I/III) ou la souche ME49 (type II), et immunosupprimées avec Cy une fois par semaine pendant un mois et après infection d’épreuve par les souches virulentes CH3 ou EGS (I/III). Les parasites récupérés sur les souris survivantes ont été soumis à l’analyse par PCR-RFLP pour confirmer la co-infection. Des anticorps spécifiques à *T. gondii* ont été analysés par ELISA. La primo infection par la souche D8 a conféré une protection contre l’infection par les souches CH3 et EGS en comparaison avec la primo infection par ME49. Le traitement Cy a causé une leucopénie significative chez les souris infectées, ce qui a favorisé probablement la réinfection après infection d’épreuve. La réinfection était associée à une augmentation du niveau d’IgA. Les souris traitées par Cy ont présenté des niveaux d’IgA significativement plus faibles après infection d’épreuve, suggérant l’implication de cet isotype sur la protection contre la réinfection. En conclusion, la sensibilité des souris BALB/c à la réinfection par *T. gondii* est liée à des différences génétiques entre les souches de parasites utilisées pour les primo infections et pour les infections d’épreuve. L’altération de l’intégrité immunitaire de l’hôte par le traitment avec la Cy a probablement compromis la protection établie préalablement par la primo infection.

## Introduction

*Toxoplasma gondii* is an obligate intracellular parasite distributed worldwide, capable of infecting all the homeothermic animals. Infection in humans is normally asymptomatic, but it can manifest itself in a severe form in cases of congenital toxoplasmosis and transmission in inmmunocompromised individuals (Dubey, 2010). The immunity acquired by primary infection with *T. gondii* was believed to be capable of preventing reinfection (Pffaf *et al.*, 2007). However, cases of congenital toxoplasmosis have been reported in infants born to immunocompetent mothers who had been infected with the parasite before conception, suggesting the occurrence of maternal reinfection during pregnancy (Elbez-Rubinstein *et al.*, 2009). Reinfection by *T. gondii* was confirmed in experimental models (Dzitko *et al.*, 2006). However, it is not yet clear whether this process is dependent on parasite genotype (Freyre *et al.*, 2004). Recently, we showed that the occurrence of experimental reinfection by recombinant strains of *T. gondii* isolated in Brazil is related not only to the genotype of the strain, but also to mouse lineage (Brandão *et al.*, 2011) and immunological alterations dependent on the time of the host’s primary infection (Brandão *et al.*, 2009). However, no studies evaluating the process of reinfection in immunosuppressed animal models are currently available.

Cyclophosphamide (Cy) has been used in the treatment of several types of cancer. Because it presents immunosuppressing properties, it has also been used in the treatment of autoimmune diseases, non-specific immunopathies, and to prevent transplant rejection (Allison, 2000; Emadi *et al.*, 2009). The major effect of Cy is on B-cells and its effects on T-cells depend on the dosage and timing of Cy administration (Allison, 2000). This study aimed to evaluate the interference of the genotypic differences in the process of reinfection by recombinant strains of *T. gondii* in Cy-immunosuppressed mouse model.

## Materials and methods

### *Toxoplasma gondii* strains

Four different *T. gondii* cystogenic strains were used in this study. The D8 (avirulent) and CH3 (virulent) strains of *T. gondii* were isolated in Brazil from a dog and a chicken, respectively (Brandão *et al.*, 2006). The EGS (highly virulent) strain was isolated in Brazil from a human with congenital toxoplasmosis (Vidigal *et al.*, 2002). Their recombinant genotypes (types I/III) were described elsewhere (Ferreira *et al.*, 2006). The ME49 (avirulent) strain was isolated from a sheep in the USA (Lunde & Jacobs, 1983) and it has a clonal genotype II.

### Experimental mice

Female Swiss mice and BALB/c mice, six-eight weeks old, weighing 18–20 g at the beginning of the experiments, were obtained from Center of Bioterism (CEBIO) of the Institute of Biological Sciences – Universidade Federal de Minas Gerais (UFMG). Swiss mice were used for parasite maintenance and to obtain tachyzoites for DNA extraction. BALB/c mice were used for leukocyte count experiments and reinfection experiments. The experiments performed in this study were approved by the Institutional Ethics Committee in Animal Experimentation (CETEA-UFMG protocol No. 038/5).

### Expression of circulating leucocytes in mice primary infected with *t. gondii* and immunosuppressed with cyclophosphamide (CY)

Brain cysts from the D8 and ME49 strains of *T. gondii* were obtained from Swiss mice previously inoculated perorally (p.o.) with five freshly prepared brain cysts. BALB/c mice were divided into six groups of six animals. Two groups were inoculated p.o. with 20 cysts of the D8 strain and two groups with 20 cysts of the ME49 strain. Two groups of naive BALB/c mice were kept as controls (Table I). After 38 days, the infection was confirmed by ELISA. 40 days after primary infection, one group infected with the D8 strain (group D8/ Cy), one group infected with the ME49 strain (group ME49/Cy) and one group of naive BALB/c mice (group Naive/Cy) were immunosuppressed weekly with 70 mg/kg/mouse of Cy (Genuxal®, Baxter Oncology GmbH, Germany). Cy was dissolved in sterile PBS pH 7.2 and intraperitoneally (i.p.) administered until the end of the experiment (Khalifa *et al.*, 2000). For leukocyte count, blood was collected from each mouse at 45, 55, 65 and 75 days after infection. Global leukocyte count was carried out using a microscope counter chamber (hemocytometer) and the values were expressed in leukocytes/mm3 of blood. Differential count was performed using Giemsa-stained blood smear. 100 leukocytes were counted and classified as basophils, eosinophils, lymphocytes, monocytes and neutrophils.

**Table I.** Mean number and standard deviation of total circulating leukocytes (expressed in cells × 103/mm3) in the blood obtained 45, 55, 65 and 75 days after infection of BALB/c mice with the D8 or ME49 strains of *Toxoplasma gondii*.

		Days after infection											
Groups		45	55	65	75	45	55	65	75	45	55	65	75
Control	Strain	D8				ME49				Naive			
	Leukocytes (10^3^/mm^3^)	5,31 ± 1,2	8,53 ± 2,7	9,0 ± 3,9	9,96 ± 4,2	5,83 ± 1,5	6,54 ± 1,3	7,23 ± 1,4	6,15 ± 1,2	8,16 ± 3,0	5,14 ± 1,0	7,69 ± 1,5	5,66 ± 0,5

Immunosuppressed	Strain	D8/Cy				ME49/Cy				Naive/Cy			
	Leukocytes (10^3^/mm^3^)	2,03 ± 0,4*	2,88 ± 0,4*	3,16 ± 0,7*	2,98 ± 0,7*	3,17 ± 0,5*	2,81 ± 0,5*	2,75 ± 0,2*	2,59 ± 0,7*	4,56 ± 1,9*	3,15 ± 1,0*	3,42 ± 0,9*	3,17 ± 0,8*

Weekly immunosuppression with cyclophosphamide (Cy) began forty days after infection, until the end of the experiment. Control groups of non-immunosuppressed mice: D8, ME49 and Naive (without infection);

* significant difference in relation to respective non-immunosuppressed control group (p < 0.05).

### Primary infection and reinfection of mice with *T. gondii*

Brain cysts from the D8 and ME49 strains of *T. gondii* were obtained as described previously. Brain cysts of the CH3 and EGS strains were obtained from Swiss mice inoculated with five brain cysts and orally-treated with sulfadiazine during ten days. BALB/c mice were divided into 18 groups of eight to ten animals (Table II). Mice were inoculated p.o. with 20 cysts of the D8 strain (groups 1–6) or 20 cysts of the ME49 strain (groups 10–15). 40 days after the primary infection, experimental groups of primary infected mice (2, 4, 6, 11, 13 and 15) were immunosuppressed weekly with Cy until the end of the experiment, as previously described. Groups of naive BALB/c mice (9 and 18) were treated weekly with Cy simultaneously, and maintained as controls. Groups 1, 3, 5, 10, 12 and 14 were kept without immunosuppression. 45 days after the primary infection, groups 1 (D8+CH3), 2 (D8+CH3/ Cy), 10 (ME49+CH3) and 11 (ME49+CH3/Cy) were challenged p.o. with 20 cysts of the CH3 strain, and groups 3 (D8+EGS), 4 (D8+EGS/Cy), 12 (ME49+EGS) and 13 (ME49+EGS/Cy) were challenged p.o. with 20 cysts of the EGS strain. Control groups 5 (D8), 6 (D8/Cy), 14 (ME49) and 15 (ME49/Cy) of the primary infected mice were maintained without challenge. Simultaneously to the challenge, naive BALB/c mice were primary infected with the CH3 strain (groups 7 and 16) and EGS strain (groups 8 and 17) and used as controls. After challenge, mortality of the animals was observed over 30 days. The animals that survived were sacrificed and their brain examined for total number of tissue cysts, and used for DNA analysis.

**Table II.** Survival, brain cysts and PCR of BALB/c mice primary infected with the D8 or ME49 strains, immunosuppressed or non-immunosuppressed with cyclophosphamide (Cy), and challenged 45 days after primary infection with the CH3 or EGS strains of *Toxoplasma gondii*.

			Reinfection		PCR-RFLP^e^ n/N (%)	
	Group^a^	Strains/Immunosuppression^b^	Survival^c^ n/N (%)	Cyst number^d^	*cS10-A6* locus	*L363* locus
BALB/c mice primary infected with D8 strain of *T. gondii*	1	D8+CH3	10/10 (100)	230,0 ± 127,4	0/10 (0)	ND
	2	D8+CH3/Cy	10/10 (100)	365,0 ± 219,9	0/10 (0)	ND
	3	D8+EGS	10/10 (100)	135,0 ± 66,9^f^	9/10 (90)	ND
	4	D8+EGS/Cy	4/10 (40)	662,5 ± 579,3^f^	2/4 (50)	ND
	5	D8	10/10 (100)	185,0 ± 91,4	ND	ND
	6	D8/Cy	10/10 (100)	140,0 ± 69,9	ND	ND
	7	CH3	0/10 (0)	NS	ND	ND
	8	EGS	0/10 (0)	NS	ND	ND
	9	Naive/Cy	10/10 (100)	0	ND	ND

BALB/c mice primary infected with ME49 strain of *T. gondii*	10	ME49+CH3	9/9 (100)	551,1 ± 270,9	ND	4/9 (44,4)
	11	ME49+CH3/Cy	7/9 (77,8)	350,0 ± 155,5	ND	5/7 (71,4)
	12	ME49+EGS	8/9 (88,9)	2543,7 ± 3646,9^g^	ND	8/8 (100)
	13	ME49+EGS/Cy	0/9 (0)	NS	ND	NS
	14	ME49	8/8 (100)	312,5 ± 138,2^g^	ND	ND
	15	ME49/Cy	7/8 (87,5)	250,0 ± 141,4	ND	ND
	16	CH3	0/9 (0)	NS	ND	ND
	17	EGS	0/9 (0)	NS	ND	ND
	18	Naive/Cy	9/9 (100)	0	ND	ND

a Experimental groups 1–18 are described on details in “Material and Methods”. The experiments were repeated twice and provided similar results.

b It indicates: strain of primary infection + strain of challenge (when performed) / Cy treatment (when performed). Naive: mice without infection.

c Number of survivors (n) out of the total number of mice challenged (N).

d Mean number of brain cysts (and standard deviation) evaluated 30 days post challenge.

e Number of mice presenting the two strains (n) (positive PCR at *cS10-A6* or *L363* locus) out of the total number of survivors after challenge (N).

f Significant difference between mice primary infected with the D8 strain and challenged with the EGS strain and mice primary infected with the D8 strain, Cy-immunosuppressed and challenged with the EGS strain, p < 0.05.

g Significant difference between mice primary infected with the ME49 strain and challenged with the EGS strain and mice primary infected with the ME49 strain, p < 0.05. NS: No survival; ND: Not done.

### Polymerase chain reaction-restriction fragment length polymorphisms (PCR-RPLF)

Genotyping was performed using *cS10-A6* and *L363* genetic markers to confirm reinfection of the mice (Ferreira *et al.*, 2006). These markers were chosen because *cS10-A6* has been shown to distinguish the D8 strain from the CH3 and EGS strains and *L363* has been shown to distinguish the strain ME49 from the CH3 and EGS strains (Ferreira *et al.*, 2006). To obtain tachyzoites for DNA extraction, the brain cysts of each mouse that survived after challenge were inoculated i.p. into the Swiss mice. Tachyzoites were harvested from the peritoneum of each mouse under aseptic conditions five to seven days after inoculation. DNA was extracted using “Wizard® Genomic DNA Purification Kit” (Promega), according to protocol described by the manufacturer. The PCR-RFLP conditions were the same as previously described (Ferreira *et al.*, 2006). For the *cS10-A6* genetic marker, amplifications were performed by using primers 5’CTGGTTACATTTTCGCCTATCA3’ and 3’CCTAGTCCAAACTAGGGCTTGA5’, producing a 341-bp fragment. PCR products were digested with restriction enzyme *Rsa*I. For the *L363* genetic marker, amplifications were performed by using primers 5’GGCTATTCGGCAAACAACAC3’ and 3’GCAATCCAGTGAGTCACCAA5’, producing a 505-bp fragment. PCR products were digested with restriction enzyme *HpyCH*4IV. The DNA banding pattern was resolved in 5 % polyacrylamide gels and silver stained. The RH88 (type I), ME49 (type II) and VEG (type III) strains were used as references.

### Enzyme-linked immunosorbent assay (ELISA)

Blood was collected from each mouse 40 days after primary infection with the D8 or ME49 strains, immediately before the beginning of immunosuppression with Cy (day = 0). Blood was also collected 30 days after challenge. Sera were tested individually for anti-*T. gondii* specific IgA, IgM and IgG (total IgG, IgG1 and IgG2a) by ELISA as previously described (Brandão *et al.*, 2009). Briefly, microplates were coated with soluble tachyzoite antigen (STAg) of the RH strain (0.5 µg/well). Sera were diluted 1:100 (IgG total) and 1:50 (IgG1, IgG2a, IgM and IgA) in PBStween- 20 at 0.05 % (PBS-T), and incubated at 37 °C for 45 min. Peroxidase-conjugated anti-mouse IgG (or IgG1, IgG2a, IgM and IgA) (SIGMA) was added to each well. The reaction was visualized with H2O2 plus ortho-phenylenodiamine and stopped with 4 N H2SO4. Absorbance was read at 490 nm on a microplate reader BIORAD model 3550. A cutoff value was calculated from the mean OD + 3 SD of eight noninfected control sera samples. Each serum sample was assayed in duplicate, taking the mean as the final result. Negative and positive controls were included on each plate.

### Statistical analysis

The statistical significance of differences between cyst numbers and absorbance means of ELISA in the different mice groups was determined by the Kruskal- Wallis non-parametric test. The leukocyte count results obtained in the different mice groups were analyzed by Kruskal-Wallis and Mann Whitney non-parametric tests. For all statistical tests mentioned above, the difference was considered statistically significant when p < 0.05.

## Results

### Circulating leukocyte count

After blood collections, the Cy-immunosuppressed mice (D8/Cy, ME49/Cy and Naive/Cy) presented a total circulating leukocyte number significantly smaller than the non-immunosuppressed control group mice (D8, ME49 and Naive) (Table I). Leukopenia occurred mainly as a result of significant reduction of lymphocytes and neutrophils (data not shown).

### Mortality and brain cysts

Challenge with the CH3 or EGS strains of *T. gondii* in mice previously infected with the D8 strain and nonimmunosuppressed with Cy (groups 1 and 3) did not lead to the death of the animals (Table II). Challenge with the CH3 strain in mice primary infected with the D8 strain and immunosuppressed with Cy (group 2) did not lead to the death of the animals, but challenge with the EGS strain in mice primary infected with the D8 strain and immunosuppressed with Cy (group 4) led to the death of six out of ten animals (60 %). The number of cysts in the brain of group 4 mice (D8+EGS/ Cy) significantly increased, compared to non-immunosuppressed and EGS strain-challenged mice (group 3). Challenge with the CH3 strain in non-immunosuppressed mice primary infected with the ME49 strain (group 10) did not lead to death of the animals (Table II). One (11.1 %) out of nine mice primary infected with the ME49 strain died when challenged with the EGS strain (group 12). Challenge with the CH3 strain in mice primary infected with the ME49 strain and immunosuppressed with Cy (group 11) led to the death of two out of nine animals (22.2 %). Challenge with the EGS strain in mice primary infected with the ME49 strain and immunosuppressed with Cy (group 13) led to the death of all (100 %) the animals. The number of brain cysts in non-immunosuppressed mice primary infected with the ME49 strain and challenged with the EGS strain (group 12), significantly increased compared to the non-challenged mice (group 14). All the mice infected only with the D8 strain (group 5) or ME49 strain (group 14) and all the mice without infection and immunosuppressed with Cy (groups 9 and 18) survived after 30 day follow-up. All mice primary infected with only the CH3 or the EGS strains (groups 7, 8, 16 and 17) died before 30 days of infection. All mice primary infected with the D8 strain and immunosuppressed with Cy (group 6) survived. Mortality occurred in one out of eight (12.5 %) mice primary infected with the ME49 strain and immunosuppressed with Cy (group 15).

### Genotyping of *T. gondii* by PCR-RFLP

**Fig. 1.**
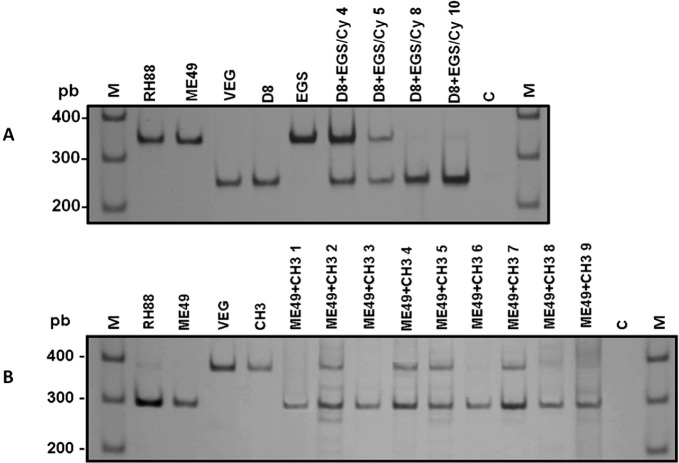
Polymerase chain reaction restriction fragment length polymorphism (PCR-RFLP) of *Toxoplasma gondii* in 5 % polyacrylamide gel silver stained to verify reinfection. A – *cS10-A6* locus with restriction endonuclease *Rsa*I; mice were primary infected with the D8 strain, immunosuppressed with Cy and challenged with the EGS strain (survivors of group 4). B – *L363* locus with restriction endonuclease *HpyCH*4IV; mice were primary infected with the ME49 strain and challenged with the CH3 strain (group 10). RH88 (Type I), ME49 (Type II) and VEG (Type III) strains were used as reference. M: molecular weight marker (Promega 100 pb); C: negative control, without DNA. The experiment was repeated twice and provided similar results.

Analysis carried out with the *cS10-A6* marker in DNA samples of *T. gondii* showed the co-existence of the D8 and EGS strains in brains of nine out of 10 (90 %) non-immunosuppressed mice (group 3) and in two out of four (50 %) survivors immunosuppressed with Cy (group 4) (Table II). CH3 strain was not detected in the brain of the D8 strain-primary infected mice (groups 1 and 2). Analysis carried out with the *L363* marker showed the co-existence of the ME49 and CH3 strains in 44.4 % (four out of nine) of the nonimmunosuppressed mice (group 10) and in 71.4 % (five out of seven) of the Cy-immunosuppressed mice (group 11). DNA analysis showed that 100 % of the non-immunosuppressed mice, primary infected with the ME49 strain were reinfected with the EGS strain (group 12) (Table II). Genotyping was not performed in group 13 (ME49+EGS/Cy) because there were no survivors. Representative results of PCR-RFLP analysis of *T. gondii* DNA obtained from mice of groups 4 and 10 are shown in Fig. 1A and Fig. 1B, respectively.

### *T. gondii* antibodies

**Fig. 2.**
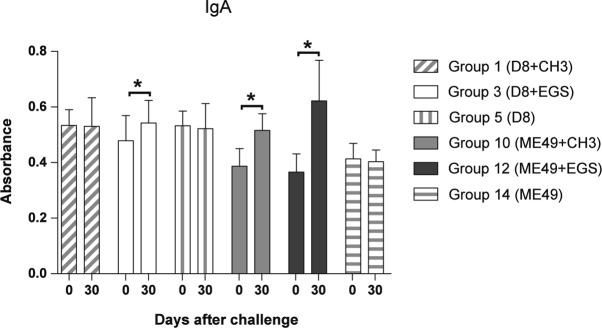
Specific IgA antibodies to *Toxoplasma gondii* detected by ELISA in sera of non-immunosuppressed BALB/c mice, primary infected with D8 strain and challenged after 45 days with the CH3 or EGS strains (groups 1 and 3, respectively) and non-immunosuppressed BALB/c mice, primary infected with the ME49 strain and challenged after 45 days with the CH3 or EGS strains (groups 10 and 12, respectively). Control groups were infected with the D8 strain (group 5) and ME49 (group 14) and non-challenged. * significant difference between sera collected before (day 0) and 30 days (day 30) after challenge, p < 0.05.

**Fig. 3.**
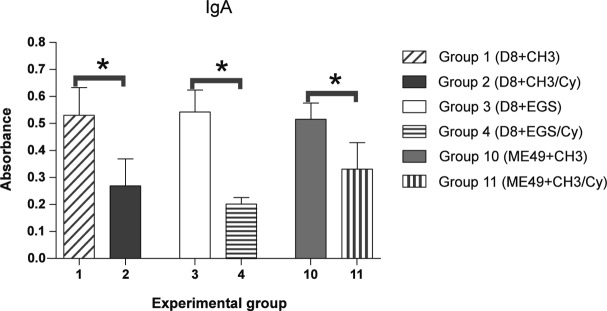
Specific IgA antibodies to *Toxoplasma gondii* detected by ELISA in BALB/c mice sera collected 30 days after challenge. * significant difference between the Cy-immunosuppressed group compared to respective non-immunosuppressed group primary infected and challenged with the same strains of *T. gondii*, p < 0.05. Experimental groups: group 1 (D8+CH3) × group 2 (D8+CH3/Cy): mice primary infected with the D8 strain and challenged with the CH3 strain; group 3 (D8+EGS) × group 4 (D8+EGS/Cy): mice primary infected with the D8 strain and challenged with the EGS strain; group 10 (ME49+CH3) × group 11 (ME49+CH3/Cy): mice primary infected with the ME49 strain and challenged with the CH3 strain.

Successful primary infection with the D8 and ME49 strains was confirmed by IgG-ELISA in all inoculated BALB/c mice. Absorbance values for IgM (data not shown) and IgA presented a significant increase (p < 0.05) on day 30 compared to day zero for non-immunosuppressed mice (groups 1, 3, 10 and 12), except for IgA in mice primary infected with the D8 strain and challenged with the CH3 strain (group 1) (Fig. 2). 30 days after challenge, mice primary infected with the D8 or ME49 strains, immunosuppressed with Cy and challenged (groups 2, 4 and 11) presented absorbance values for IgM (data not shown) and IgA significantly smaller (p < 0.05) compared to nonimmunosuppressed mice (groups 1, 3 and 10, respectively) (Fig. 3). Absorbance values for IgG, IgG1, and IgG2a did not present significant alterations on day 30, in relation to day zero. No significant decrease was verified in these antibodies when immunosuppressed and non-immunosuppressed mice were compared (p > 0.05) (data not shown).

## Discussion

All BALB/c mice primary infected with the virulent CH3 or EGS strains of *T. gondii* died, however, when infection by these strains was preceded by primary infection with the non-virulent D8 or ME49 strains, in the absence of immunosuppression, mortality rate is considerably reduced. These results show that primary infection elicits an adaptative immune response capable of protecting the immunocompetent animals against the virulent strain used in the challenge. PCR-RFLP showed that there was no reinfection by the CH3 strain after primary infection with D8 strain, but it indicated the coexistence of the D8 and EGS strains in the brain of challenged mice. These results confirm a previous study which had shown that mice were reinfected with the EGS strain at 45 days post primary infection with the D8 strain, but they were not reinfected with the CH3 strain (Brandão *et al.*, 2009). PCR-RFLP showed reinfection by the CH3 and EGS strains after primary infection with ME49 strain. Previous studies had reported an increase in the number of brain cysts following reinfection of mice with other strain of *T. gondii* (Araújo *et al.*, 1997; Brandão *et al.*, 2009). In this study, nonimmunosuppressed mice primary infected with the ME49 strain, had a significant increase in the number of brain cysts after challenge with the EGS strain, followed by reinfection and consequent colonization of their brains by the secondary strain. However, increase in the number of brain cysts cannot be considered a reinfection marker, since not all groups with confirmed reinfection by PCR-RFLP had this increase.

Besides evaluating the participation of the *T. gondii* genotype in the process of reinfection, this study verified whether Cy-immunosuppression favors reinfection. Cy was chosen based on the literature, as this drug has been utilized to cause immunosuppression in experimental models and it has favorable characteristics to this purpose (Allison, 2000; Khalifa *et al.*, 2000; Emadi *et al.*, 2009). All mice primary infected with the D8 strain and immunosuppressed with Cy survived after 30 days follow-up. Only one mouse primary infected with the ME49 strain and treated with Cy died. Cy-immunosuppression did not lead to an increase in the number of brain cysts in primary infected mice. Such results indicate that Cy, at the dosage and intervals applied, was not capable of reactivating the latent chronic infection. Reactivation of infection can involve rupture of cysts and release of parasites into the infected tissues. Histopathological analysis may later prove whether the use of Cy at doses higher than those described here may reactivate primary infection with *T. gondii*.

In mice primary infected with the D8 strain, Cy-immunosuppression before challenge with the EGS strain significantly increased the mortality rate (from zero to 60 %) and brain cysts number. Similarly, when mice primary infected with the ME49 strain were immuno- suppressed and challenged with the CH3 strain, mortality increased from zero to 22.2 %. In mice primary infected with the ME49 strain and challenged with the EGS strain, mortality also increased significantly (from 11.1 % to 100 %), when challenge was preceded by Cy-immunosuppression. These data show that the Cy-treatment increases the susceptibility of mice after challenge with a virulent strain of *T. gondii*. To our knowledge, this is the first study that evaluates the process of reinfection by *T. gondii* in Cy-immunosuppressed mice.

Primary infection with the D8 strain conferred more protection against challenge with the CH3 and EGS strains than primary infection with the ME49 strain, both in Cy-immunosuppressed and non-immunosuppressed mice. Also, primary infection with the D8 strain was capable of preventing reinfection with the CH3 strain, even after immunosuppression, but primary infection with the ME49 strain allowed reinfection by the two strains. The D8, CH3 and EGS strains belong to the recombinant genotype I/III, predominant in Brazil, while the ME49 strain belongs to the clonal type II genotype, frequently found in Europe and in the USA (Ferreira *et al.*, 2006). Thus, the greater genotypic difference between the ME49 strain and the CH3 and EGS strains may have likely been a determinant factor for the low protection conferred by primary infection. Other authors had previously emphasized the importance of the genotypic differences in the process of reinfection (Araújo *et al.*, 1997; Dzitko *et al.*, 2006). However, these authors carried out studies using clonal strains. In another study, mice were primary infected with the PRU strain (clonal type II) and challenged with the IPP-2002-URB strain (atypical genotype, commonly found in South America), confirming the occurrence of reinfection (Elbez-Rubinstein *et al.*, 2009). Our results corroborate the authors’ hypothesis that the immunity acquired against European strains may not protect against reinfection by strains of a different genotype. Challenge with the EGS strain was responsible for greater rates of reinfection and mortality among the primary infected mice, compared with the CH3 strain. The EGS strain (Lethal dose 100 % = 1 tachyzoite) is more virulent than the CH3 strain (Lethal dose 100 % ≥ 10 tachyzoites) (Ferreira *et al.*, 2006). Thus, the reinfection process may also be associated to virulence of the strain used in the challenge, since the tachyzoites of virulent strains have a greater capacity of invading cells, crossing biological barriers and multiplying in the intracellular medium (Barragan & Sibley, 2002).

Leukopenia is a common adverse effect of Cy and has been used as immunosupressing therapy guide (Allison, 2000; Emadi *et al.*, 2009). In our work, leukopenia occurred mainly due to the simultaneous reduction in the number of neutrophils and lymphocytes. During the early phase of the *T. gondii* oral infection, neutrophils rapidly migrate at the site of infection and participate in the recruitment and activation of other immune cells such as macrophages and dendritic cells. These cells are important in protecting the host against *T. gondii* infection, because they trigger an initial immune response that limits tachyzoite replication and produce cytokines that induce a Th1 phenotype (Buzoni-Gatel *et al.*, 2006). CD4+ and CD8+ T lymphocytes are the main cellular types involved in resistance of the host to *T. gondii* infection, because they act synergistically, providing a protective immunity that allows the survival of the host during the chronic infection (Pfaff *et al*., 2007). The reduction in the number of leukocytes observed in this study is likely related to a greater susceptibility of immunosuppressed BALB/c mice to reinfection by *T. gondii*. Previously, we have shown that greater susceptibility of C57BL/6 mice to reinfection by *T. gondii* when compared to BALB/c mice is probably related to lower amount of IFN-γ and IL-10 in prime-infected C57BL/6 mice (Brandão *et al.*, 2011). So, further studies on cellular immune response are required to investigate which lymphocyte population is being affected by Cy and which cytokine profile is predominant in the different organs of the infected mice treated with Cy, thus helping to clarify the immune mechanism responsible for the increase in susceptibility.

A previous study reported a significant increase in the levels of anti-*T. gondii* IgG after reinfection of mice (Dzitko *et al.*, 2006). In our study, the production of IgG antibodies and its sub-classes, IgG1 and IgG2a, did not undergo significant alterations after reinfection. A significant increase in the levels of IgA antibodies was observed 30 days after challenge. IgA is an important element of the mucosal immune response against *T. gondii* oral infection and is related to the acute toxoplasmosis (Buzoni-Gatel *et al.*, 2006). Significant increase of IgM and IgA to *T. gondii* was previously reported after experimental reinfection in mice (Hassan *et al.*, 1999; Brandão *et al.*, 2009), corroborating the results found in our study. However, IgM increase is not a reliable reinfection marker since mice challenged with the CH3 strain after primary infection with the D8 strain were not reinfected but presented an increase of this immunoglobulin.

Levels of IgG, IgG1 and IgG2a did not undergo alterations after Cy administration. However, IgA production was significantly smaller in the Cy-treated mice 30 days after challenge. These results were expected because Cy has a deleterious effect on B lymphocytes after a single dose of the drug, being capable of inhibiting the antibodies production in mice against different antigens (Rollinghoff *et al.*, 1977; Doherty, 1981). The immunoglobulins can limit the multiplication of *T. gondii* by activation of the complement, promoting opsonization and increasing the phagocytosis by the macrophages (Correa *et al.*, 2007). Some studies emphasize the importance of the antibodies response in protecting the host against infection by *T. gondii* (Kang *et al.*, 2000; Johnson & Sayles, 2002). A previous study demonstrated that mortality of the Cy-treated mice infected by *T. gondii* was considerably reduced after passive immunization with serum obtained from mice chronically infected by the same strain of the parasite (Hafizi & Modabber, 1978). The smaller production of IgM and IgA antibodies found in our study was probably one of the determinant factors for the higher reinfection rates found after challenge in the Cy-treated animals. Further studies on Cy-immunosuppressed mice are necessary to investigate specific inhibition of the synthesis of IgM and IgA, but not of IgG.

It can be concluded that susceptibility of BALB/c mice to reinfection by *T. gondii* is associated to the genotypic differences between primary infection and the challenge strains. The increase in susceptibility to reinfection after Cy-immunosuppression is associated to decrease of IgA and leukocytes number what can likely compromise the protection previously established by the primary infection. Taking into account these results, it is necessary to establish primary prevention among the patients chronically infected by *T. gondii* and submitted to Cy-immunosuppression treatment, due to possible increase in susceptibility to reinfection.
